# Prevalence of Positive Childhood Experiences Among Adults — Behavioral Risk Factor Surveillance System, Four States, 2015–2021

**DOI:** 10.15585/mmwr.mm7317a3

**Published:** 2024-05-02

**Authors:** Robert Sege, Elizabeth A. Swedo, Dina Burstein, Maria V. Aslam, Jennifer Jones, Christina Bethell, Phyllis Holditch Niolon

**Affiliations:** ^1^Institute for Clinical Research and Health Policy Studies, Tufts Medical Center, Boston Massachusetts; ^2^Division of Violence Prevention, National Center for Injury Prevention and Control, CDC; ^3^Division of Injury Prevention, National Center for Injury Prevention and Control, CDC; ^4^Prevent Child Abuse America, Chicago, Illinois; ^5^Johns Hopkins University Bloomberg School of Public Health, Baltimore, Maryland.

**Summary**

**What is already known about this topic?**

Positive childhood experiences (PCEs), children’s experiences of safe, stable, and nurturing relationships and environments, promote healthy child development and adult mental and relational health and buffer against negative impacts of adverse childhood experiences.

**What is added by this report?**

This population-based study presents PCE prevalence among U.S. adults in four states. Approximately one half of adults (53.1%) reported six to seven PCEs; 12.2% reported two or fewer. PCEs were lower among lesbian, gay, and bisexual adults and higher among respondents with higher income and educational attainment.

**What are the implications for public health practice?**

Integration of PCEs data collection into public health surveillance can guide approaches to promote well-being and reduce health disparities.

## Abstract

Positive childhood experiences (PCEs) promote optimal health and mitigate the effects of adverse childhood experiences, but PCE prevalence in the United States is not well-known. Using Behavioral Risk Factor Surveillance System data, this study describes the prevalence of individual and cumulative PCEs among adults residing in four states: Kansas (2020), Montana (2019), South Carolina (2020), and Wisconsin (2015). Cumulative PCE scores were calculated by summing affirmative responses to seven questions. Subscores were created for family-related (three questions) and community-related (four questions) PCEs. The prevalence of individual PCEs varied from 59.5% (enjoyed participating in community traditions) to 90.5% (adult in respondents’ household made them feel safe), and differed significantly by race and ethnicity, age, and sexual orientation. Fewer non-Hispanic Black or African American (49.2%), non-Hispanic Alaska Native or American Indian (37.7%), and Hispanic or Latino respondents (38.9%) reported 6–7 PCEs than did non-Hispanic White respondents (55.2%). Gay or lesbian, and bisexual respondents were less likely than were straight respondents to report 6–7 PCEs (38.1% and 27.4% versus 54.7%, respectively). A PCE score of 6–7 was more frequent among persons with higher income and education. Improved understanding of the relationship of PCEs to adult health and well-being and variation among population subgroups might help reduce health inequities.

## Introduction

Positive childhood experiences (PCEs), children’s experience of having safe, stable, nurturing relationships and environments, promote healthy child development and adult mental and relational health* (*1*). PCEs also buffer the effects of adverse childhood experiences (*1*) and reduce the prevalence of adult health risk behaviors, such as smoking or unhealthy alcohol use (*2*). Previous reports have looked at single states (*1*,*2*) or selected populations (*3*). This report, presenting the weighted prevalence of individual and cumulative PCEs in four states that included PCE questions in their Behavioral Risk Factor Surveillance System (BRFSS), is the largest study of the prevalence of PCEs among U.S. adults to date.

## Methods

### Data Source

BRFSS is an annual, state-based telephone survey of health-related behaviors and chronic health conditions of noninstitutionalized adults collected from all 50 states and the District of Columbia (*4*). This study analyzed BRFSS data from four states that included seven identical, PCE questions added by the states on their survey: Kansas (2020), Montana (2019), South Carolina (2020), and Wisconsin (2015). The survey response rates ranged from 45.0% to 51.5%; response rate to PCE questions ranged from 97.3% to 99.6%. PCE survey items were adapted from the Child and Youth Resilience Measure (*1*,*5*) and included three family items^†^ and four community items.^§^ The survey used a five-level Likert-type scale and directed the respondents to “refer to the time before you were 18 years of age.” Responses were scored as present if the respondent answered “Often,” “Very Often,” “Most of the time,” or “All of the time.” After accounting for missing values, the final analytic sample included 24,893 respondents. Participants who were not living in the survey administration state at the time of the survey (249; 0.8%) or who were missing data for more than two PCE items (3,728; 12%) were excluded. This activity was reviewed by CDC, deemed not research, and was conducted consistent with applicable federal law and CDC policy.^¶^

### Data Analysis

Cumulative PCE scores were calculated by summing affirmative responses to each of the seven PCE types and then categorized into groups 0–2, 3–5, or 6–7 (*1*). Family and community subscores were created by summing affirmative responses to the family and community PCE items. Weighted prevalence estimates and 95% CIs were calculated for individual PCEs and cumulative PCE scores in total, by state, and by sociodemographic characteristic (sex, age, race and ethnicity, annual household income, educational attainment, employment status, and sexual orientation). Nonoverlapping CIs were used to assess statistically significant prevalence differences between sociodemographic categories. Weighted family and community subscore means and 95% CIs were compared using t-tests. All analyses accounted for survey design using recommended weights, and complex survey procedures were conducted in SAS (version 9.4; SAS Institute) and verified in Stata (version 18; StataCorp).

## Results

Prevalence of individual PCEs ranged from 59.5% (enjoyed participating in community traditions) to 90.5% (adult in respondent’s household who made them feel safe) (Table 1). Prevalence of individual PCEs varied significantly by race and ethnicity, age group, and sexual orientation. For example, 47.4% of participants self-identifying as bisexual reported that they “felt a sense of belonging in high school” compared with 73.1% of participants who identified as straight.

**TABLE 1:** Positive childhood experiences among adults — Behavioral Risk Factor Surveillance System, four U.S. states, 2015–2020

Characteristic (no. of respondents)	Weighted % (95% CI)*						
	Adult made you feel safe and protected	Felt sense of belonging in high school	Felt supported by friends	At least two nonparent adults took an interest	Felt family stood by you	Enjoyed community traditions	Felt able to talk to family
**Total (20,916)**	**90.5 (89.9–91.1)**	**71.8 (70.8–72.7)**	**80.3 (79.5–81.2)**	**71.8 (70.9–72.8)**	**61.0 (60.0–62.0)**	**59.5 (58.5–60.6)**	**82.1 (81.3–82.9)**
**Sex**							
Female (11,357)	89.6 (88.7–90.4)	70.5 (69.2–71.7)	79.6 (78.5–80.7)	72.3 (70.9–73.5)	60.1 (58.7–61.5)	61.6 (60.2–63.0)	79.8 (78.6 −81.0)
Male (9,559)	91.5 (90.6–92.3)	73.1 (71.8–74.5)	81.1 (79.8–82.3)	71.4 (69.9–72.8)	62.0 (60.4–63.4)	57.4 (55.8–58.9)	84.5 (83.4–85.6)
**State**							
Kansas (4,456)	88.7 (87.4–89.9)	69.7 (67.9–71.4)	80.6 (79.0–82.1)	70.9 (69.1–72.4)	64.2 (62.3–66.0)	65.7 (63.9–67.6)	82.9 (81.3–84.3)
Montana (5,627)	89.5 (88.4–90.5)	69.5 (67.9–71.0)	80.7 (79.3–82.0)	71.1 (69.6–72.5)	56.1 (54.5–57.7)	59.8 (58.2–61.4)	79.3 (77.9–80.6)
South Carolina (5,950)	91.1 (90.0–92.0)	73.1 (71.5–74.6)	79.6 (78.1–80.9)	73.9 (72.6–75.5)	61.2 (59.4–62.9)	61.2 (59.4–62.9)	81.5 (80.0–82.9)
Wisconsin (4,883)	91.2 (90.0–92.3)	72.2 (70.3–74.0)	80.6 (79.0–82.1)	70.5 (68.6–72.3)	60.1 (58.1–62.1)	54.4 (52.4–56.5)	82.9 (81.4–84.3)
**Income**							
<$15,000 (1,545)	83.7 (80.8–86.2)	55.3 (51.2–59.3)	65.2 (61.3–68.9)	61.0 (57.0–64.8)	53.7 (49.8–57.4)	49.6 (46.6–52.5)	69.8 (65.9–73.4)
$15,000–$24,999 (2,721)	86.5 (84.6–88.3)	61.8 (59.9–64.7)	71.8 (69.1–74.4)	62.3 (59.3–65.2)	55.1 (52.2–58.0)	50.0 (47.1–53.0)	73.4 (70.8–75.9)
$25,000–$34,999 (1,954)	89.4 (87.3–91.3)	69.6 (66.3–72.7)	75.4 (72.2–78.3)	69.2 (66.0–72.3)	59.0 (55.6–62.4)	55.7 (52.6–59.2)	80.7 (77.7–83.4)
$35,000–$49,999 (2,767)	90.1 (88.1–91.8)	71.3 (68.4–73.9)	80.6 (78.1–82.8)	69.7 (66.8–72.5)	59.3 (57.6–62.1)	55.7 (52.8–58.7)	82.1 (79.7–84.2)
>$50,000 (9,036)	93.1 (92.2–93.8)	78.0 (76.7–79.3)	86.4 (85.3–87.5)	77.8 (76.5–79.1)	64.5 (63.0–66.0)	65.7 (64.2–67.2)	86.8 (85.8–87.9)
**Education**							
Less than high school (1,177)	83.7 (80.7–86.4)	47.9 (43.5–52.5)	60.7 (56.6–64.7)	54.5 (50.3–58.7)	53.3 (49.1–57.5)	45.8 (41.7–50.1)	71.3 (67.4–75.0)
High school diploma or GED (5,746)	89.8 (88.6–90.9)	71.5 (69.7–73.2)	80.0 (78.4–81.6)	68.6 (66.7–70.4)	62.0 (60.1–63.9)	54.6 (52.7–56.5)	81.0 (79.5–82.5)
Some college (6,192)	90.9 (89.8–91.9)	73.1 (71.5–74.7)	82.0 (80.6–83.3)	73.1 (71.5–74.8)	59.6 (57.7–61.3)	59.1 (57.3–60.9)	81.7 (80.3–83.1)
College degree (7,752)	93.5 (92.6–94.3)	78.5 (77.1–79.9)	86.1 (84.9–87.3)	80.5 (79.2–81.8)	64.7 (63.1–66.2)	70.9 (69.4–72.4)	88.0 (86.9–89.0)
**Employment status**							
Employed (10,385)	91.5 (90.6–92.2)	72.2 (70.9–73.5)	81.8 (80.6–82.8)	73.3 (72.0–74.6)	62.2 (60.8–63.5)	59.1 (57.6–60.5)	83.5 (82.4–84.6)
Unemployed (821)	84.2 (80.3–87.4)	60.5 (55.4–65.3)	71.5 (66.6–75.9)	65.9 (61.0–70.5)	52.6 (47.6–57.6)	52.3 (47.2–57.3)	72.7 (67.8–77.0)
Unable to work (1,358)	80.6 (77.1–83.6)	54.1 (50.1–58.1)	64.2 (60.4–67.8)	58.1 (54.2–61.9)	49.0 (45.1–52.8)	47.3 (43.4–51.2)	65.7 (61.8–69.4)
Other (8,232)	92.0 (91.0–92.8)	76.9 (75.4–78.3)	82.8 (81.5–84.1)	73.1 (71.6–74.6)	63.1 (61.5–64.7)	64.6 (63.0–66.2)	84.6 (83.3–85.8)
**Race and ethnicity** ^†^							
AI/AN (530)	86.4 (78.4–91.8)	56.2 (47.8–64.3)	71.3 (63.4–78.1)	69.9 (61.8–77.0)	51.2 (42.9–59.4)	58.4 (49.9–66.5)	76.9 (70.0–82.6)
Asian (129)	85.2 (74.1–92.0)	70.4 (58.3–80.1)	82.6 (71.8–89.8)	64.8 (52.0–75.8)	55.8 (43.3–67.7)	60.2 (47.9–71.3)	73.3 (60.9–82.8)
Black or African American (1,651)	91.7 (89.5–93.5)	71.6 (68.0–75.0)	75.0 (71.8–78.0)	73.8 (70.4–77.0)	59.9 (56.2–63.5)	58.0 (54.2–61.6)	79.3 (75.9–82.3)
White (17,212)	91.0 (90.4–91.7)	72.8 (71.8–73.8)	82.2 (81.3–83.0)	72.7 (71.7–73.7)	61.5 (60.4–62.6)	60.5 (59.4–61.6)	83.5 (82.6–84.3)
Hispanic or Latino (567)	86.6 (82.9–89.7)	67.0 (61.2–72.3)	70.8 (65.2–75.9)	56.9 (51.0–62.5)	58.6 (53.0–64.1)	51.4 (45.6–57.2)	74.1 (68.9–78.7)
Other race (164)	84.0 (73.3–90.9)	56.8 (43.0–69.7)	72.6 (58.2–83.5)	67.5 (52.1–79.9)	57.8 (45.1–69.6)	55.4 (42.0–68.0)	79.9 (69.4–87.5)
Multiracial (419)	83.2 (76.8–88.1)	59.5 (51.6–67.0)	70.8 (63.4–77.3)	70.9 (63.2–77.6)	61.6 (54.3–68.4)	49.9 (42.3–57.4)	76.0 (69.2–81.7)
**Age group, yrs**							
18–24 (1,259)	90.5 (88.0–92.4)	68.0 (64.4–71.1)	78.9 (75.7–81.9)	73.2 (69.7–76.5)	61.0 (57.4–64.5)	50.3 (46.6–54.0)	79.4 (76.2–82.3)
25–34 (1,899)	88.9 (87.0–90.5)	64.2 (61.2–67.2)	79.1 (76.5–81.4)	69.3 (66.2–72.1)	59.8 (56.7–62.8)	52.6 (49.4–55.7)	78.1 (75.4–80.6)
35–44 (2,296)	89.4 (87.5–91.0)	69.1 (66.3–71.7)	77.6 (75.0–80.0)	72.8 (70.0–75.4)	60.1 (57.2–63.0)	58.4 (55.5–61.3)	79.9 (77.5–82.2)
45–54 (2,930)	89.1 (87.4–90.6)	72.1 (69.8–74.3)	80.5 (78.5–82.4)	71.9 (69.5–74.1)	59.4 (56.9–61.8)	61.5 (58.9–63.9)	82.4 (80.5–84.3)
55–64 (4,469)	91.6 (90.4–92.8)	73.0 (71.3–74.9)	80.4 (78.7–82.1)	71.5 (69.6–73.4)	60.5 (58.4–62.5)	61.7 (59.6–63.8)	83.0 (81.4–84.5)
≥65 (8,063)	92.4 (91.5–93.2)	79.2 (77.8–80.5)	83.5 (82.2–84.8)	72.3 (70.8–73.8)	63.9 (62.3–65.5)	66.7 (65.1–68.3)	86.6 (85.4–87.7)
**Sexual orientation**							
Bisexual (412)	77.9 (72.0–82.9)	47.4 (40.9–53.9)	70.3 (64.2–75.8)	58.6 (51.9–64.9)	39.9 (33.9–46.3)	45.3 (39.0–51.7)	69.8 (63.5–75.3)
Gay or lesbian (268)	89.5 (84.0–93.2)	55.5 (46.5–64.2)	70.6 (62.2–77.7)	61.6 (52.3–70.0)	49.5 (40.6–58.5)	47.1 (38.3–56.1)	76.2 (68.7–82.3)
Straight (19,485)	91.2 (90.6–91.8)	73.1 (72.1–74.0)	81.1 (80.2–82.0)	73.0 (72.0–73.9)	62.0 (60.9–63.0)	60.5 (59.4–61.5)	82.9 (82.0–83.7)
Another sexual orientation (234)	71.4 (62.1–79.2)	48.4 (39.0–57.9)	63.5 (53.9–72.1)	46.7 (37.6–56.2)	38.8 (30.0–48.4)	39.0 (30.3–48.4)	63.3 (53.4–72.2)

**Abbreviations:** AI/AN = American Indian or Alaska Native; GED = general educational development certificate.

* Reflects noninstitutionalized adults (aged ≥18 years) in Kansas, Montana, South Carolina, and Wisconsin.

^†^ Persons of Hispanic or Latino (Hispanic) origin might be of any race but are categorized as Hispanic; all racial groups are non-Hispanic.

Overall, 53.1% of respondents reported 6–7 PCEs, 34.7% reported 3–5, and 12.2% reported 0–2 (Table 2). Prevalence of low PCE scores (0–2) was higher among women (13.2%) than among men (11.2%).

**TABLE 2:** Positive childhood experiences among adults — Behavioral Risk Factor Surveillance System, four U.S. states, 2015–2020

Characteristic (no. of respondents)	No. of positive childhood experiences % (95% CI)		
	0–2	3–5	6–7
Total (20,916)*	**12.2 (11.5–12.9)**	**34.7 (33.7–35.7)**	**53.1 (52.1–54.1)**
**Sex**			
Female (11,357)	13.2 (12.3–14.2)	33.6 (32.2–35.0)	53.2 (51.8–54.6)
Male (9,559)	11.2 (10.2–12.2)	35.9 (34.4–37.4)	53.0 (51.4–54.5)
**State**			
Kansas (4,456)	11.3 (10.1–12.6)	34.0 (32.3–35.9)	54.6 (52.7–56.5)
Montana (5,627)	13.8 (12.7–15.0)	35.9 (34.4–37.5)	50.3 (48.7–51.9)
South Carolina (5,950)	11.8 (10.7–13.1)	34.6 (32.9–36.3)	53.6 (51.9–55.3)
Wisconsin (4,883)	12.7 (11.4–14.1)	35.0 (33.0–36.9)	52.3 (50.3–54.3)
**Income**			
<$15,000 (1,545)	23.3 (19.9–27.0)	38.9 (35.2–42.7)	37.8 (34.2–41.6)
$15,000–$24,999 (2,721)	19.7 (17.5–22.1)	39.5 (36.6–42.5)	40.8 (38.0–43.7)
$25,000–$34,999 (1,954)	13.7 (11.6–16.1)	37.7 (34.3–41.3)	48.6 (45.1–52.0)
$35,000–$49,999 (2,767)	12.9 (11.0–15.1)	36.1 (33.3–39.0)	51.0 (48.1–53.9)
>$50,000 (9,036)	7.9 (7.1–8.8)	30.5 (29.1–32.0)	61.6 (60.0–63.1)
**Education**			
Less than high school (1,177)	25.5 (22.2–29.3)	43.6 (39.5–47.8)	30.9 (27.2–34.9)
High school diploma or GED (5,746)	12.9 (11.7–14.3)	36.6 (34.7–38.5)	50.5 (48.6–52.4)
Some college (6,192)	11.3 (10.2–12.4)	35.4 (33.7–37.2)	53.3 (51.5–55.2)
College degree (7,752)	7.4 (6.6–8.3)	28.3 (26.8–29.8)	64.3 (62.7–65.9)
**Employment status**			
Employed (10,385)	11.3 (10.4–12.3)	34.0 (32.7–35.4)	54.7 (53.2–56.1)
Unemployed (821)	19.7 (15.9–24.1)	41.4 (36.5–46.5)	38.9 (34.2–43.8)
Unable to work (1,358)	26.1 (22.8–29.7)	40.3 (36.5–44.3)	33.5 (30.1–37.1)
Other (8,232)	9.5 (8.5–10.5)	33.3 (31.7–35.0)	57.2 (55.5–58.8)
**Race and ethnicity** ^†^			
AI/AN (530)	16.9 (11.9–23.4)	45.4 (37.1–53.9)	37.7 (30.3–45.7)
Asian (129)	15.9 (8.7–27.4)	37.6 (26.8–49.9)	46.4 (34.4–58.9)
Black or African American (1,651)	11.7 (9.4–14.3)	39.1 (35.5–42.8)	49.2 (45.6–52.9)
White (17,212)	11.6 (10.9–12.4)	33.1 (32.1–34.2)	55.2 (54.1–56.4)
Hispanic or Latino (567)	17.3 (13.6–21.7)	43.8 (38.1–49.6)	38.9 (33.5–44.6)
Other race (164)	15.8 (8.9–26.4)	45.0 (32.1–58.6)	39.2 (27.8–51.8)
Multiracial (419)	18.5 (13.5–24.8)	37.6 (30.3–45.4)	43.9 (36.7–51.3)
**Age group, yrs**			
18–24 (1,259)	12.0 (9.8–14.7)	40.3 (36.7–44.0)	47.7 (44.0–51.3)
25–34 (1,899)	14.4 (12.4–16.7)	39.3 (36.3–42.5)	46.3 (43.2–49.4)
35–44 (2,296)	14.6 (12.6–16.8)	32.1 (29.4–35.0)	53.3 (50.3–56.2)
45–54 (2,930)	13.6 (12.0–15.5)	32.6 (30.3–35.0)	53.8 (51.3–56.3)
55–64 (4,469)	11.2 (10.0–12.6)	34.6 (32.6–36.6)	54.2 (52.1–56.3)
≥65 (8,063)	9.1 (8.2–10.1)	32.0 (30.5–33.6)	58.9 (57.2–60.5)
**Sexual orientation**			
Bisexual (412)	23.6 (18.5–29.7)	48.9 (42.5–55.4)	27.4 (22.3–33.2)
Gay or lesbian (268)	20.9 (14.3–29.4)	41.0 (32.6–50.1)	38.1 (29.8–47.1)
Straight (19,485)	11.5 (10.8–12.2)	33.8 (32.8–34.9)	54.7 (53.6–55.8)
Another sexual orientation (234)	31.9 (23.6–41.5)	43.3 (34.3–52.8)	24.8 (17.8–33.3)

**Abbreviations:** AI/AN = American Indian or Alaska Native; GED = general educational development certificate.

* All estimates are weighted to reflect noninstitutionalized adults (aged ≥18 years) in Kansas, Montana, South Carolina, and Wisconsin.

^†^ Persons of Hispanic or Latino (Hispanic) origin might be of any race but are categorized as Hispanic; all racial groups are non-Hispanic.

The proportion of respondents with high PCE scores (6–7) varied by race and ethnicity, age, employment status, and sexual orientation. In particular, 37.7% of non-Hispanic American Indian or Alaska Native adults reported high PCE scores, compared with 55.2% of non-Hispanic White adults. Gay or lesbian and bisexual respondents were less likely to report high PCE scores (38.1% and 27.4%, respectively) than were those who identified as straight (54.7%). Respondents with income ≥$50,000 were more likely to report 6–7 PCEs (61.6%) than were those with income <$15,000 (37.8%). Similarly, respondents with a college degree were more likely than those who had not completed high school to report 6–7 PCEs (64.3% versus 30.9%) (Table 2).

### Subscore Results

Overall, the mean family PCE subscore was 2.3 (out of 3); the mean community PCE subscore was 2.8 (out of 4) (Table 3). The mean community subscore was lower among respondents with household income <$15,000 than among those with income ≥$50,000 (2.2 versus 3.1; p<0.001) and was lower among persons with less than a high school education (2.0) than among those with a college degree (3.2; p<0.001). The mean community subscore was higher among employed respondents (2.8) than among those who were unemployed (2.5) or unable to work (2.2) (p<0.001); the mean was higher among respondents who identified as straight (2.8) than among those who described themselves as gay or lesbian (2.3), bisexual (2.2), or another sexual orientation (2.0) (p<0.001).

**TABLE 3:** Positive childhood experience subscores* among adults, by demographic characteristic — Behavioral Risk Factor Surveillance System, four U.S. states, 2015–2020

Characteristic (no. of respondents)	Family subscore		Community subscore	
	Mean (95% CI)	p-value^†^	Mean (95% CI)	p-value^†^
**Total (20,916)**	**2.3 (2.3–2.4)**	**—**	**2.8 (2.7–2.8)**	**—**
**Sex**				
Female (11,357)	2.3 (2.2–2.3)	<0.001	2.8 (2.7–2.8)	0.669
Male (9,559)	2.4 (2.3–2.4)	Ref	2.8 (2.7–2.8)	Ref
**State**				
Kansas (4,456)	2.4 (2.3–2.4)	Ref	2.8 (2.8–2.9)	Ref
Montana (5,627)	2.2 (2.2–2.3)	<0.001	2.8 (2.7–2.8)	0.053
South Carolina (5,950)	2.3 (2.3–2.4)	0.318	2.8 (2.8–2.9)	0.926
Wisconsin (4,883)	2.3 (2.3–2.4)	0.571	2.7 (2.7–2.8)	0.023
**Income**				
<$15,000 (1,545)	2.1 (2.0–2.1)	Ref	2.2 (2.1–2.4)	Ref
$15,000–$24,999 (2,721)	2.1 (2.1–2.2)	0.110	2.4 (2.3–2.5)	0.055
$25,000–$34,999 (1,954)	2.3 (2.2–2.4)	<0.001	2.7 (2.6–2.8)	<0.001
$35,000–$49,999 (2,767)	2.3 (2.2–2.4)	<0.001	2.8 (2.7–2.9)	<0.001
>$50,000 (9,036)	2.4 (2.4–2.5)	<0.001	3.1 (3.0–3.1)	<0.001
**Education**				
Less than high school (1,177)	2.1 (2.0–2.2)	Ref	2.0 (1.9–2.1)	Ref
High school diploma or GED (5,746)	2.3 (2.3–2.4)	<0.001	2.7 (2.7–2.8)	<0.001
Some college (6,192)	2.3 (2.3–2.4)	<0.001	2.9 (2.8–2.9)	<0.001
College degree (7,752)	2.5 (2.4–2.5)	<0.001	3.2 (3.1–3.2)	<0.001
**Employment status**				
Employed (10,385)	2.4 (2.3–2.4)	Ref	2.8 (2.8–2.9)	Ref
Unemployed (821)	2.1 (2.0–2.2)	<0.001	2.5 (2.3–2.6)	<0.001
Unable to work (1,358)	1.9 (1.8–2.0)	<0.001	2.2 (2.1–2.3)	<0.001
Other (8,232)	2.4 (2.3–2.4)	0.384	2.9 (2.9–3.0)	0.001
**Race and ethnicity** ^§^				
AI/AN (530)^†^	2.1 (2.0–2.3)	0.007	2.5 (2.3–2.8)	0.005
Asian (129)	2.1 (1.9–2.4)	0.064	2.7 (2.4–3.0)	0.456
Black or African American (1,651)	2.3 (2.2–2.4)	0.120	2.7 (2.7–2.8)	0.029
White (17,212)	2.4 (2.3–2.4)	Ref	2.8 (2.8–2.9)	Ref
Hispanic or Latino (567)	2.2 (2.1–2.3)	0.002	2.4 (2.2–2.5)	<0.001
Other race (164)	2.2 (2.0–2.5)	0.270	2.5 (2.2–2.8)	0.028
Multiracial (419)	2.2 (2.0–2.4)	0.069	2.5 (2.3–2.7)	<0.001
**Age group, yrs**				
18–24 (1,259)	2.3 (2.2–2.4)	Ref	2.7 (2.6–2.8)	Ref
25–34 (1,899)	2.3 (2.2–2.3)	0.352	2.6 (2.5–2.7)	0.310
35–44 (2,296)	2.3 (2.2–2.3)	0.765	2.7 (2.7–2.8)	0.368
45–54 (2,930)	2.3 (2.2–2.3)	0.882	2.8 (2.8–2.9)	0.010
55–64 (4,469)	2.4 (2.3–2.4)	0.389	2.8 (2.8–2.9)	0.006
≥65 (8,063)	2.4 (2.4–2.5)	0.004	3.0 (2.9–3.0)	<0.001
**Sexual orientation**				
Bisexual (412)	1.9 (1.7–2.0)	<0.001	2.2 (2.0–2.4)	<0.001
Gay or lesbian (268)	2.1 (2.0–2.3)	<0.001	2.3 (2.1–2.6)	<0.001
Straight (19,485)	2.3 (2.3–2.4)	Ref	2.8 (2.8–2.9)	Ref
Another sexual orientation (234)	1.7 (1.5–1.9)	<0.001	2.0 (1.7–2.2)	<0.001
**Positive childhood experience score**				
0–2 (2,310)	0.7 (0.7–0.8)	Ref	0.5 (0.5–0.6)	Ref
3–5 (6,835)	2.0 (2.0–2.1)	<0.001	2.2 (2.1–2.2)	<0.001
6–7 (11,771)	2.9 (2.8–2.9)	<0.001	3.7 (3.7–3.8)	<0.001

**Abbreviations:** AI/AN = American Indian or Alaska Native; GED = general educational development certificate; Ref = referent group.

* All estimates are weighted to reflect noninstitutionalized adults (aged ≥18 years) in four states (Kansas, Montana, South Carolina, and Wisconsin).

^†^ p-values are based on t-test.

^§^ Persons of Hispanic or Latino (Hispanic) origin might be of any race but are categorized as Hispanic; all racial groups are non-Hispanic.

## Discussion

This study is the largest population-based assessment of PCEs among U.S. adults to date. Experiencing PCEs is common among adults and varies by sociodemographic characteristics. An estimated one half of adults report at least six of seven measured PCEs, and approximately one in eight persons report 2 or fewer. A higher PCE score was observed among employed adults and those with higher educational attainment and income. In addition to associations with adult mental and relational health (*1*), longitudinal data from Australia suggests that PCEs lead to improved mental health and academic attainment in adolescence (*6*). Further exploration is needed to understand differences in the prevalence of PCEs across educational attainment, employment, and income subgroups. CDC’s ACEs Prevention: Resource for Action** and Tufts Medical Center’s HOPE National Resource Center^††^ offer practical suggestions for interventions to bolster PCEs and prevent adversity. PCEs occur within families, schools, and the community. Public policies that promote parent-infant bonding, such as paid family leave and home visiting, and free or low-cost access to out-of-school time programming, including arts and athletics, might help to reduce observed inequities in PCE scores.

### Limitations

The findings in this report are subject to at least four limitations. First, population-based estimates of PCEs in these four states might have limited generalizability nationally. Second, BRFSS questions measure a limited set of PCEs that do not include all postulated PCEs (*6*). Third, social desirability and recall biases might reduce the accuracy of self-reported PCEs. Specific PCEs might have been experienced differently by certain groups, contributing to the sociodemographic differences observed. Finally, cross-sectional data cannot demonstrate causality.

### Implications for Public Health Practice

Assessment of PCEs could be added to other public health data collection efforts. This action has the potential to improve understanding of determinants of overall well-being, which could in turn guide public health interventions that might support structures that promote PCEs (*7*,*8*) and reduce inequities in adult health and well-being. Further study is needed to examine the effects of PCEs on adult health as well as interactions among PCEs, adverse childhood experiences, and health outcomes.

Individual PCEs and PCE scores might provide applicable metrics for public health surveillance (*9*) and for evaluating interventions to improve child and adult well-being. Public health approaches might improve access to community-level PCEs, which are associated with higher adult economic status and educational attainment. The National Council of State Legislatures, for example, cited efforts to improve early childhood education and fund family resources as policy levers to promote resilience (*10*). Inequities in PCEs might be a focus for public health interventions, especially given the previously reported effects of PCEs on protecting mental and relational health (*1*). Given that fewer racial, ethnic, and sexual minority adults felt a sense of belonging in high school, efforts to promote a sense of belonging for all high school students might be helpful. Further research might address the possible lifelong effects of PCEs, including the observed association of high PCE scores and higher educational and income attainment.

## Footnotes

* Relational health is defined as “the ability to develop and maintain safe, stable, nurturing relationships with other individuals and to engage in social activities.”

† Felt able to talk to their family about their feelings; felt their family stood by them during difficult times; felt safe and protected by an adult in their home.

§ Enjoyed participating in community traditions; felt a sense of belonging in high school (not including those who did not attend school or were homeschooled); felt supported by friends; had at least two nonparent adults who took genuine interest in them.

††
htpps://positiveexperience.org
